# Does carbon footprint reduction impair mechanical properties and service life of concrete?

**DOI:** 10.1617/s11527-022-02090-9

**Published:** 2022-12-29

**Authors:** Kiran Ram, Marijana Serdar, Diana Londono-Zuluaga, Karen Scrivener

**Affiliations:** 1 Department of Materials, Faculty of Civil Engineering, University of Zagreb, 10000 Zagreb, Croatia; 2 Laboratory of Construction Materials, École Polytechnique Fédérale de Lausanne, 1015 Lausanne, Switzerland

**Keywords:** Low clinker cement, Chloride penetration, Service life, Environmental impact assessment, Sustainability

## Abstract

The aim of this study is to evaluate how much the changes in the concrete mix design, which enable carbon footprint reduction, are impacting mechanical properties and predicted service life of concrete structure. The starting point of this study was concrete mix used in a recent reinforced concrete Pelješac Bridge in the Adriatic. In the first round of experiments the amount of cement in this initial mix was significantly lowered, without jeopardising workability of the mix. In the second round, the main part of the cement was substituted with the combination of fly ash and limestone or calcined clay and limestone. All supplementary cementitious materials used were sourced in the region of the structure. The calcined clays used in this study were collected locally and found to have a low kaolin content. On all mixes fresh and mechanical properties were tested to ensure that the requested equal or better workability and mechanical stability were reached. Furthermore, on each mix chloride migration was tested to evaluate the resistance of mix to chloride penetration. All mixtures were evaluated based on the overall performance considering mechanical, durability, and carbon footprints. The results indicate that the total cement content had a significant effect on durability and thus service life. The bridge mix design was determined to be ‘over designed,’ as all alternative mixes achieved a similar or higher sustainability index with lower amount of cement.

**Supplementary Information:**

The online version contains supplementary material available at 10.1617/s11527-022-02090-9.

## Introduction

Concrete is known to be the most sought-after building material in the world. Among all other industries, the cement industry has a significant carbon footprint of about 8% [1]. Consequently, concrete has a significant impact on the environment due to the cement industry. Current projections indicate that by 2050, approximately 18 billion tonnes of concrete will be required for worldwide construction, leading to an increase in carbon dioxide emissions from cement production. In recent decades, several attempts have been made to manufacture ecologically friendly concrete while also enhancing the efficiency of clinker [2–7].

Performance-based concrete design is one of the most straightforward and effective methods for enhancing clinker efficiency. In accordance with the 2016 united nations environment program standards (UNEP-SBCI) [8], clinker efficiency must be enhanced by optimizing mix design with industry participation. The primary objective of mix design optimization is to prevent overdesigning of concrete, which can lower the amount of cement in concrete and its carbon impact. Standards prescribing a minimum cement content and a maximum water content for concrete to achieve the required characteristics are the most significant barrier to reducing the cement content of concrete [9, 10]. Therefore, such a rigid, prescriptive design contributes to the reluctance of engineers to use alternative approaches based on concrete performance.

Other obstacles include concerns about long-term durability due to lower binder content, loss of workability due to insufficient cement paste volume, and problems with the bond between the steel and cement matrix [11]. Because of these technical concerns, engineers are unwilling to take the risk of incorporating the reduced binder content into their mix design, even though most of these concerns have been addressed. For example, workability of concrete is maintained by using a high-range water reducer without increasing the w/b ratio [12–14] or without adding any water or cement to the system. There are some studies that have looked at the durability of concrete with a lower binder content. In these studies, it was reported that the cement content can be lowered without sacrificing durability, especially maintaining equal penetrability of the concrete [13, 15–17]. Due to the higher proportion of aggregate compared to cement paste, the permeability of the concrete can be significantly reduced. In addition, the cement in the concrete may not be fully hydrated [18], especially in a concrete with a very low water–cement ratio (*w*/*c*). The extent of cement hydration is limited due to the lack of water in these systems, and the unhydrated cement particles end up acting as fillers [19]––which can be trail by inexpensive filler such as limestone without significant environmental or economic impact [20, 21].

The second most applied strategy of increasing clinker efficiency is introducing supplementary cementitious materials (SCMs), such as fly ash (FA) and blast furnace slag (BFS), for low CO_2_ cements. Low-CO_2_ cements containing SCM have shown improved mechanical strength, durability, and sustainability, resulting in blended cements being the most produced cements worldwide [22–24] However, given the changes in the energy sector and the greening of other industries, these usual SCMs will not be available on a scale to meet the global demand for concrete and cement. Therefore, other materials need to be considered as potential cement substitutes. Recent research studies have confirmed that kaolin clays can be used as a pozzolanic material, ensuring good performance of concrete and offering economic and environmental advantages [25–27]. However, most of these studies focused on high-purity kaolin clays, metakaolin, and clays with a kaolin content of at least 40%, which are difficult to find worldwide. Therefore, it is important to consider the possibility of using lower-grade clays (with less than 40% kaolinite), especially where high-purity clays are in short supply.

The objective of this work was to determine if reducing cement content and substituting it with locally available SCMs would affect the overall performance of the concrete structure in a marine environment. The effect of changes at the material level on the overall performance of structures is of paramount importance for wider acceptance and application of these solutions in practice. Numerous published studies attempt to comprehend the material properties resulting from variations in concrete formulation, which is undeniably essential and frequently leads to significant advances in cement research. The intent of this manuscript, on the other hand, is to demonstrate the impact of these changes in material properties on the overall performance of structures. The manuscript is based on a case study that takes the actual mix design for a built bridge, attempts to produce alternative mixes using locally available materials, and evaluates their overall performance in the structure. Therefore, the idea of this study is to analyse the limit to which it is possible to reduce the environmental impact of materials without compromising the performance of the structure.

The performance criteria were taken from a real concrete structure of Peljesac Bridge, a recently completed monumental structure designed for a 100-year service life in an aggressive Adriatic marine environment [28]. Beginning with the actual concrete mix for the bridge, fifteen mixtures were examined, ranging from a reduction in cement content to a high-volume cement substitute. The high-volume replacement was achieved using a combination of limestone––fly ash and limestone—calcined clay, with all materials readily available in the cement plant region. The performance of the materials and their individual contribution to the overall performance are beyond the scope of this study. Rather, the purpose is to demonstrate whether the mechanical stability and service life of concrete structures are affected by the selection of mixes with lower environmental impacts. Therefore, the overall performance of all mixes in this study included a combination of the following parameters: (1) mechanical properties based on compressive strength, (2) service life calculated based on chloride diffusion coefficient and Fick’s second law, (3) economic impact based on material price, (4) environmental impact based on global warming potential (GWP), and (5) environmental impact based on embodied energy.

## Materials and methods

### Binder composition

**Fig. 1:**
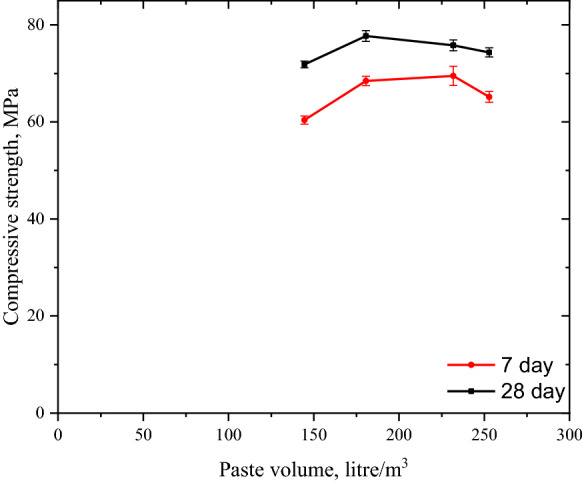
Variation in compressive strength with respect to paste volume

The bridge mix was designed using blended cement with slag addition, CEM II B–S in accordance with EN 196-1. The primary material in all alternative binder systems was Portland cement (CEM I 425R) according to EN 196-1. The physical and chemical composition, as well as the mean particle size of all cementitious materials were determined using X-ray fluorescence method and laser diffraction technique (please see Table 1; Fig. 1 in additional documents). All materials were obtained locally in the region of the cement plant. The chemical admixture employed in the mixture is based on polycarboxylate ether, and the solid content was determined as 35%, using the ASTM C484 method.

**Table 1:** Five performance indices for estimation of overall performance indicator

Index (I)	Weighting (w)	Equation
Embodied carbon index (*I*_EC_)	3	$I_{{{\text{EC}}}} = \frac{{{\text{GWP}}\;{\text{of}}\;{\text{reference}}\;{\text{mixture}}}}{{{\text{GWP }}\;{\text{of }}\;{\text{selected}}\;{\text{ mixture}}}}$
Service life index (*I*_SL_)	3	$I_{{{\text{SL}}}} = \frac{{{\text{Service }}\;{\text{life}}\;{ }\left( {{\text{in }}\;{\text{years}}} \right){ }\;{\text{of }}\;{\text{reference }}\;{\text{mixture}}}}{{{\text{Service }}\;{\text{life }}\;\left( {{\text{in }}\;{\text{years}}} \right)\;{\text{ of}}\;{\text{ selected}}\;{\text{ mixture}}}}$
Cost index (*I*_C_)	3	$I_{{\text{C}}} = \frac{{{\text{Cost }}\;{\text{of }}\;{\text{m}}^{{3}} \;{\text{ concrete }}\;{\text{using }}\;{\text{reference }}\;{\text{mixture}}}}{{{\text{Cost }}\;{\text{of }}\;{\text{m}}^{{3}} \;{\text{ concrete}}\;{\text{ using}}\;{\text{ selected }}\;{\text{mixture}}}}$
Embodied energy index (*I*_E_)	1	$I_{{\text{E}}} = \frac{{{\text{Embodied}}\;{\text{ energy}}\;{\text{ of}}\;{\text{ reference}}\;{\text{ mixture}}}}{{{\text{Embodied }}\;{\text{energy}}\;{\text{ of }}\;{\text{selected}}\;{\text{ mixture}}}}$
Strength Index (*I*_S_)	1	$I_{{\text{S}}} = \frac{{f_{{{\text{ck}}}} \; {\text{of }}\;{\text{reference}}\;{\text{ mixture}}}}{{f_{{{\text{ck}}}} \;{\text{of}}\;{\text{ selected}}\;{\text{ mixture}}}}$

Two clays from distinct sources were employed in the investigation, and both had a lower amount of kaolinite. The kaolinite content was determined using the thermogravimetric analysis method [29]. Clay labelled as CC1 contains 14% kaolin and clay labelled as CC2 contains 18% kaolin. Fly ash was obtained from the Tuzla thermal power plant in Bosnia and Herzegovina. The fly ash and raw clay were dried for 24 h at 60 ± °C to remove all moisture. Following the drying process, they were ground for 30 s in a laboratory ball mill (each batch of 300 g material). Finally, the clays were calcined for an hour at 800 °C in a high-temperature laboratory furnace according to the procedure described in [30]. To ensure homogeneity, the ternary binder systems were pre-mixed prior to mixing.

### Mixture details

The performance indicators were adopted from the bridge design. The targeted strength and slump fixed 50 MPa and 80–120 mm respectively. Throughout the service life the bridge will be exposed to harsh marine environment, already proven by other major reinforced concrete bridges in Croatia [31]. It is therefore that the bridge mix was designed with splash zone durability in mind, which means that diffusion coefficients should be kept to a minimum.

Two bridge mixes were prepared denoted BD435 and BD340. In BD435, the whole mix design was derived from the real mix (binder content, water/binder ratio, and aggregate amount), whereas in BD340, the binder content was reduced while maintaining the same water/binder ratio. All other mixes in the study followed equivalent mix design method. Initially, the cement type in the bridge mix was replaced with CEM I, to optimize the binder content and achieve the desired strength and workability. At this stage, mixtures with varying binder contents ranging from 350 to 200 kg/m^3^ were prepared using CEM I cement with a *w*/*b* ratio of 0.40. Thereafter, the binder content and *w*/*b* ratio were adjusted based on the fresh properties and compressive strength to avoid the out-flow performance of strength. Next, binder content was fixed to the chosen optimum of 340 kg/m^3^ and *w*/*b* to 0.4 on which cement was substituted by fly ash and limestone or calcined clay and limestone. The cement replacement level of blended mixture varied from 30 to 45%. The combination of calcined clay/fly ash and limestone powder was in the ratio of 2:1 by total replacement volume of clinker. To achieve the maximum packing of aggregate, all aggregates were arranged based on the modified Andersson curve [32]. With the optimum packing of aggregate the paste volume can be reduced, at the same time increasing the workability [33]. In the next round, the amount of cement was further decreased, increasing at the same time water content. In this round, mixtures with varying binder contents ranging from 300 to 250 kg/m^3^ were prepared using CEM I cement with a *w*/*b* ratio of 0.45. Here chosen binder content was 300 kg/m^3^ and *w*/*b* to 0.45 for high volume cement substitution with fly ash/calcined clay and limestone. The details of all mixtures were given in additional documents (see Table 2).

**Table 2:** Fresh properties of bridge mixtures and alternative mixtures

Mixture	Paste volume, l/m^3^	Slump, mm	Temperature, ^o^C	Wet density, kg/m^3^	Air content, %
BD435	310.6	105	24.2	2482.2	2.4
BD340	242.4	105	24.1	2539.9	3.1
C350	252.9	90	26.9	2461.3	2.9
C340	246.03	90	24.3	2512.1	3.5
C300	231.80	90	27.8	2502.2	3.2
C250	180.60	80	27.8	2513.3	3.2
C200	144.50	78	28.9	2541	1.5
FA40	246.42	100	24.3	2512.1	2.1
LC2-45	243.65	90	24.9	2505.1	2.8
C300	232.40	100	22.3	2490.8	2.9
C250	215.42	95	24.2	2501.1	3.2
FA40	246.42	95	23.8	2498.9	3.4
FA30	242.08	95	25.3	2361.2	3.1
LC1-45	243.65	95	22.3	2395.3	2.8
LC1-45	243.5	95	23.3	2341.3	2.7

### Preparation of concrete, fresh properties, and compressive strength

All the mixes were prepared and tested in fresh state according to EN 12350. Specimens were demoulded 24 h after casting and then cubes were transferred to the humidity chamber (relative humidity maintained more than 95% and temperature of 20 ± 1 °C) until testing day. In designing the experimental plan, an engineering approach was used, employing standardized methods, methods normally used in practice. Since the basic idea of the work was to show the effects of cement reduction on a real case in a real environment, the methods and concrete age at which the tests were performed were the same for all mixes and conformed to standards. Although further environmental savings would be possible if the performance of the materials were considered at a later age (for example 56 or 90 days), this rigorous and conservative approach was nevertheless used to clearly demonstrate potential savings from changes in concrete formulations.

Three concrete specimen of 15 cm × 15 cm × 15 cm were prepared to measure the compressive strength after 7 and 28 days of curing as per EN 12390-3 standard. Cylindrical specimen of 20 cm height and 10 cm diameter were prepared to test the chloride penetration resistance of the mixtures. After the 28 days of curing, the cylindrical specimens were split diametrically into three slices with thickness of 5 cm for the experiment.

### Chloride penetration

The chloride ingress resistance was expressed in terms of chloride migration coefficient, which was determined by nord test build 492 [34]. After the specified test duration, the specimen was splatted diametrically, and were sprayed with silver nitrate solution. The chloride penetration depth, *x*_*d*_, was visible as whitish colour. Then the non-steady state migration coefficients (*D*_nssm_) were determined based on the Nernst–Plank equation.

1 $$D_{{{\text{nssm}}}} = \frac{{{\text{RT}}}}{{z{\text{FE}}}} \cdot \frac{{x_{{\text{d}}} - \alpha \sqrt {x_{{\text{d}}} } }}{t}.$$

### Service life estimation

In this study, the calculation of service life of the reinforced structure included the corrosion initiation and propagation time. The initiation time was calculated based on the Fick’s 2nd law of diffusion [35, 36] as a time needed for the critical chloride content (Clth) to penetrate through a cover depth (*d*) and initiate the corrosion of reinforcement [37]. Therefore, the time to initiate the corrosion depends on the surface chloride concentration (Cl_s_), chloride diffusion coefficients (*D*_cl_) and the ageing coefficients (*m*). Therefore, service life of structure is written as a function of each above-mentioned parameter.

2 $${\text{Service}}\;{\text{life}}\;{\text{of}}\;{\text{a}}\;{\text{structure}} = f_{n} { }\left( {d,\,{\text{Clth}},\,m,\,D_{{{\text{cl}}}} ,\,{\text{Cl}}_{{\text{s}}} } \right)$$

In this study, a reinforcement square column of 250 mm width and 50 mm cover depth was taken as the structural member for the determination of service life. The location of the bridge was taken as the location of Peljesac Bridge, Croatia. The chloride threshold value was taken as 0.05% (by weight of concrete) and the maximum surface chloride content was 0.8% by weight of binder. The cumulative probability of structural failure was taken as 0.5 for all mixture. The ageing coefficient was assumed as 0.6 regardless of the mix. Pillai et al. [38] reported that ageing coefficients for fly ash and limestone-calcined clay cement are in the range of 0.5–0.7, and for Portland cement less than 0.20. At the same time, chloride thresholds were found to be lower in the blended system compared to the Portland cement system. The use of a higher aging coefficient for the blended system and a lower one for the Portland cement mix would result in a more significant decrease in the chloride diffusion coefficient with aging for the blended system compared to the Portland cement mix. However, the use of lower chloride thresholds for the blended system would result in a more rapid onset of corrosion compared to the Portland cement mix. Since the aging factor and chloride threshold were not measured for these particular alternative binders used in the present study, the maximum values for these two parameters were taken for all mixes [39]. However, for a more accurate calculation, the actual values of the aging factor and chloride thresholds should be measured.

Previous investigations on these and related systems found that chloride migration produces higher values than bulk diffusion. Since chloride migration was measured for all systems, a correlation factor was used to compare the two approaches.

3 $$D_{{{\text{nssm}}}} = k \times D_{{{\text{cl}}}}$$

The value of ‘*k*’ determined experimentally from several previous studies by the authors was taken as 1.69 [40].

### Evaluation of environmental impact and material cost

The environmental impact and materials costs (*A*_total_) were calculated for one cubic meter of concrete, which were total sum of each individual values of materials multiplied (*x*_*i*_) by corresponding quantity (*q*_*i*_) in one cubic meter of concrete.

4 $$A_{{{\text{total}}}} = { }\sum \left( {q_{i} { } \times x_{i} } \right).$$

#### Environmental impact

The environmental impact assessment considered the environmental impact of producing concrete constituents from raw materials, transporting the raw materials, and finally the production of one cubic meter of concrete. The construction phase, service phase and demolition phase were not considered in the analysis since they were presumed to be equal for all systems. The present work environmental impact was expressed in terms of embodied carbon and energy as per the guidelines in ISO 14040. Embodied carbon is expressed as global warming potential (GWP). GWP is a measure of the total emission of greenhouse gases (equivalent to CO_2_) to the atmosphere. Each material was analyzed up to the point of manufacturing, excluding the sources of raw materials. The conversion parameters for each material used to calculate the total embodied carbon/energy are given in additional documents (see Table 3), along with their corresponding references. For mixes with calcined clay and limestone (LC2 blend) mixtures, it was assumed that they were pre-mixed and used as a single binder material [41]. The emission data for the transportation and production were taken from Simapro data base [42], as well as from the literature and product data sheets [12, 43–48] (for exact numbers used, please see Table 3 in Supporting Information). The production of concrete was assumed to occur at the bridge site, and the average distance between the production plant of each constituent and concrete plant was assumed to be 100 km. The embodied carbon for the transportation was taken as 0.131 kg eq. CO_2_/kg [42].

**Table 3:** Total material cost of each mixture

Mixture	Clinker	Fly ash/calcined clay + limestone	Aggregate	Water	Superplasticizer	Total cost in Euro per m^3^
BD435	40.40	–	18.70	0.14	4.26	63.49
BD340	31.62	–	20.60	0.11	5.71	58.03
C340	33.66	–	20.38	0.11	3.81	57.96
FALS40	20.19	6.12	20.08	0.11	5.71	52.15
LC2-45	18.51	6.88	20.04	0.11	6.67	52.21
C300	29.70	–	21.96	0.11	4.20	55.97
FALS40	17.82	5.40	20.49	0.11	5.04	48.85
FALS30	20.79	4.05	20.57	0.11	5.46	50.98
LC1-45	16.33	6.06	20.53	0.11	5.88	48.91
LC1-40	17.82	5.40	20.53	0.11	5.46	49.32

#### Material cost

The total cost of a cubic meter of concrete was calculated as a sum of individual material costs multiplied by their quantity per cubic meter of concrete. Each material cost was expressed in price (Euro) per kilogram, and data were gathered from local suppliers. Fly ash and calcined clay are still not commercially available, and as a result, a similar price was assumed for fly ash-limestone and LC2 blends. The prices of each material used in the study are given in additional documents (see Table 4 in Supporting Information).

### Overall performance in terms of sustainability

Reducing the binder content or using alternative binders in concrete without considering the impact on durability would be a failure in terms of sustainability. Moreover, in today’s world, economic advantages will be to the detriment of a more environmentally friendly concrete. Therefore, the sustainability of any concrete mix is a function of these parameters. To allow comparison of the mixes, the values of all parameters were converted into a single value indicating the sustainability of each mix. The overall performance of each mix was expressed using two different approaches: (1) sustainability factor (*β*) proposed by Muller et al. [46], and (2) five-performance index (*I*_5*P*_) based on the study by Yu et al. [49].

#### Sustainability factor (*β*)

Muller et al. proposed a sustainability factor to evaluate the concrete mixture in terms of durability and environmental impact. The factor is determined by following relationship [46]:

5 $${\text{Sustainability}}\,{\text{factor}},\,\beta = \frac{{f_{{{\text{ck}}}} { } \times t_{{{\text{SL}}}} }}{{{\text{Embodied }}\,{\text{arbon }}\left( {{\text{GWP}}} \right)}}$$

where *f*_ck_ is 28-day compressive strength of the mixture, *t*_SL_ is the service life of the structure and embodied carbon is expressed as global warming potential.

#### Five performance index

This approach considers five distinct factors and weights them differently. For example, a global sustainability of concrete is determined more by its life span, embodied energy, and cost than by its strength. The concept is derived from a study [49], and is then modified by incorporating the service life factor. While the first approach (see Sect. 2.7.1) is relatively simple, incorporating additional factors and influences in the second approach may provide additional insight into an overall sustainability of proposed concrete mix. Additionally, this factor would indicate whether the mixture is compromising any means of sustainability when the cement content is significantly reduced. The overall performance indicator was calculated based on five different performance indices given in Table 1.

After determining each index, the overall performance indicator called the five-performance index (*I*_5*P*_) is calculated based on the following relationship:

6 $$I_{5P} = \frac{{\mathop \sum \nolimits_{i = 5} \left( {I_{i} { } \times w_{i} } \right){\text{of}}\,{\text{selected}}\,{\text{mixture}}}}{{\mathop \sum \nolimits_{i = 5} \left( {I_{i} { } \times w_{i} } \right){\text{of }}\,{\text{reference }}\,{\text{mixture}}}}$$

where *I*_*i*_ are the five different indices and *w*_*i*_ is the weight of each index presented in Table 1.

## Results and discussion

### Evolution of strength

The fresh properties of all mixes are given in the Table 2. The reduction in the total binder content significantly affected the workability of the mixture. For instance, C200 mixture was totally unacceptable even with high amount of superplasticizer due to the very low amount of paste in the system [50]. Also, the workability retention considerably reduced for C200 and C250. However, all the mixes surpassed the target strength of 50 MPa.

The effect of paste reduction on compressive strength is depicted in Fig. 1. The strength increased from mix C350–C300 and then to C250. The tendency of compressive strength to increase as paste content decreases is related to the decrease in the interfacial transition zone (ITZ), which surrounds aggregates. An ITZ has a more fragile structure than bulk hydrated cement and is more susceptible to mechanical loads. In addition, Jones et al. reported that a reduction in the cement paste (which is highly porous) and an increase in aggregate make the mixture less porous, thereby enhancing the physical contact between aggregates. Even with reduced binder content, densified interfacial transition zones (ITZ) could help to balance the strength [51–53].

Although all mixes satisfied criteria for compressive strength, due to the workability retention the binder content of 300 kg/m^3^ was chosen as a base reference for the alternative cementitious systems. Additional to this, the binder content of 340 kg/m^3^ was also used as a base reference to compare the overall performance of mixes with high volume cement substitution.

**Fig. 2:**
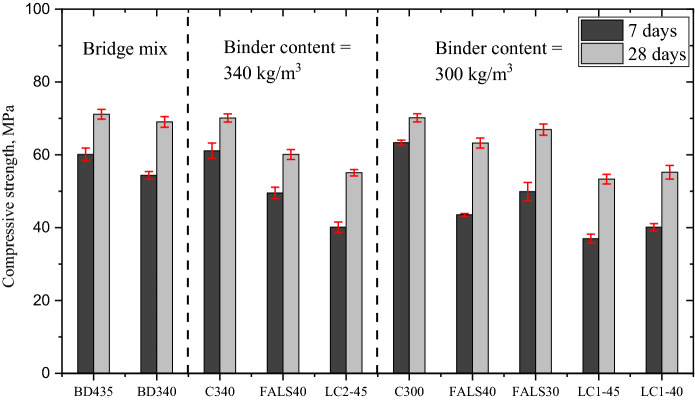
Compressive strength of all the mixture

The compressive strength of the mixes that satisfied the targeted compressive strength after 7 and 28 days is depicted in Fig. 2. In Fig. 6, the C300 and C340 were taken as the reference mixes for corresponding alternative binder system. Compressive strengths of BD435 and BD340 were comparable, and a 22% decrease in cement content had no discernible effect on compressive strength. In general, decreasing the cement content of concrete at same *w*/*b* results in increased aggregate content, which has a detrimental effect on the workability of the mixture and enhances compressive strength performance [54]. In this case study, the issue of workability was resolved through the use of appropriate chemical admixtures and optimized aggregate packing [17].

It is visible that the FALS40 is a promising mixture, capable of achieving at least 80% strength when compared to BD435. The addition of limestone in the system with fly ash and cement resulted in increased compressive strength even at 40% of cement replacement and even at early ages, contrary to the fly ash-Portland cement mixture [55]. Although the performance of the calcined clay mixture was slightly lower than that of fly ash, compressive strength still exceeded 50 MPa at 28 days with a high level of cement replacement. The pozzolanic reaction kinetics and strength development of limestone-calcined clay are highly dependent on kaolinite [56], which may explain why the compressive strength is lower than that of the FALS mix. Despite the lower kaolinite content, the compressive strength of concrete incorporating both clays were found to be satisfactory and met current performance criteria.

### Chloride penetration

**Fig. 3:**
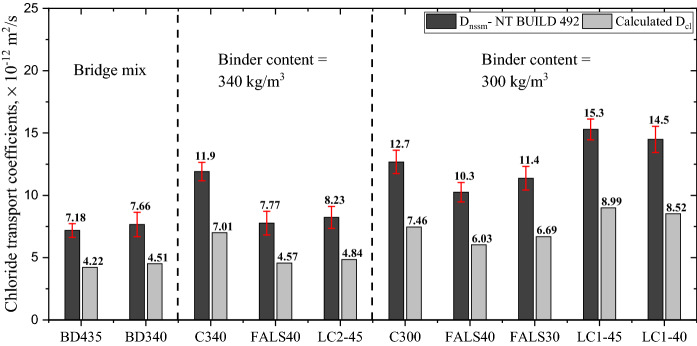
Chloride migration coefficients (*D*_nssm_) of all mixture and chloride diffusion values (*D*_cl_) calculated using the relation *D*_nssm_ = 1.69 × *D*_cl_

The durability performance of each mixture was evaluated by measuring non-stead state chloride migration coefficient (*D*_nssm_). This coefficient was then transferred into chloride diffusion coefficient shown in Fig. 3 by using the Eq. 2. Values of chloride diffusion coefficient were used to calculate the service life of each mixture in the following section.

In contrast to the compressive strength, the chloride penetration resistance of alternative binders was found to be dependent on the binder content and type of binder. Among all the mixtures, BD435 demonstrated the highest resistance to chlorides. Clearly, the bridge mix exhibits the least penetration due to the presence of blast furnace slag in cement itself. As the secondary hydrates formed, they densified the microstructure and inhibited the penetration of the chloride ion, lowering the migration coefficients [55]. However, in BD340, the 95 kg/m^3^ reduction in the total binder content did not result in a significant decrease in chloride penetration resistance, and the same compressive strength was achieved. Furthermore, when in the system with 340 kg/m^3^ of cement part of the cement was substituted with fly ash and limestone or calcined clay and limestone, a significant improvement in chloride resistance was achieved. Finally, when the binder content was further decreased from 340 to 300 kg/m^3^, the performance against chloride ingress significantly declined. In the systems with 300 kg/m^3^ of cement even the substitution of cement with fly ash and calcined clay did not results in a significant improvement in requested resistance and the resistance remained lower than that of the bridge mix design. Nevertheless, chloride migration coefficient of system with 300 kg/m^3^ and fly ash and limestone were still lower than that of 340 kg/m^3^ of cement, which is important to highlight.

Comparing bridge mix BD425 and BD340, where the only difference is 20% reduction in cement content, it can be observed that the mix with 340 kg/m^3^ achieved similar value of chloride transport. Furthermore, similar values of chloride transport were obtained when additional 40–45% of cement is substituted with fly ash and limestone and calcined clay and limestone, demonstrating that the reduction in cement content and substitution of clinker up to 45% has little effect on chloride penetration. In other words, overdesign in cement content is not required to improve the durability of concrete. However, the binder content of 300 kg/m^3^ demonstrated poorer performance than BD435, indicating that there is a reduction limit beyond which concrete structures would be negatively affected.

### Service year determination

**Fig. 4:**
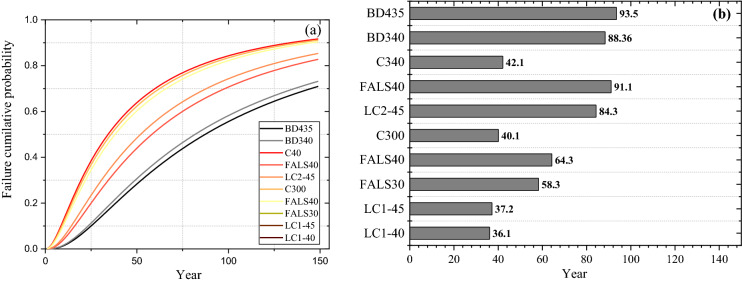
**a** Cumulative probability curves **b** Service life in year of all the mixtures

The service year of each mixture was calculated by the method explained in Sect. 2 and the cumulative probabilistic curve illustrated in the Fig. 4a, b give the values of service life at cumulative failure probability = 0.5.

The service life is calculated as the sum of the corrosion initiation and propagation times, with the propagation time for all the mixes assumed to be six years. In comparison to BD435, the FALS40 mixture had a similar expected service life. The addition of 21% binder content to BD435 had no effect on service life when compared to BD340––due to the similar chloride penetration resistance. Therefore, the reduction of cement content in the bridge design had no effect on its service life. Despite the shorter service life of C300 and C340, the addition of alternative binders with the same binder content increased their service life, with the fly ash-limestone mixture exhibiting the greatest improvement. In the case of calcined clay, the proportion of kaolinite and binder affects the service life; LC2-45 exhibited a significantly longer service life than LC1-45. The difference in life span of each mixture demonstrates that the binder type has a crucial role in durability performance of concrete, rather than the amount of cement.

### Environmental impact assessment and materials cost

#### Embodied carbon and embodied energy:

**Fig. 5:**
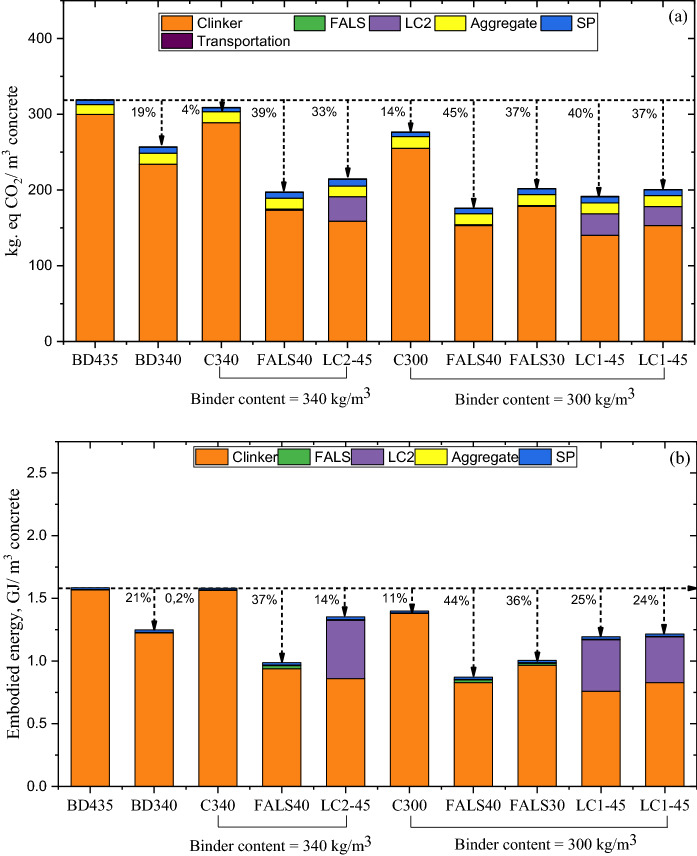
**a** Global warming potential, and **b** embodied energy of all mixtures compared to BD435

Environmental impact indicators (global warming potential and embodied energy) are illustrated in Fig. 5a, b, respectively. Additionally, the percentage of reduction compared to BD435 is clearly indicated in figures.

The figure demonstrated that the clinker component accounted for more than 70% of the carbon and energy embodied in 1 m^3^ concrete. As a result, reducing the binder content and substituting with alternative binders can create a significant reduction of the overall environmental impact of concrete. Additionally, the FALS mixture demonstrated the greatest reduction in carbon footprint in this case. In the case of calcined clay, the GWP was found to be reduced by between 30 and 40%, but the embodied energy was just 15–25%. Although the embodied energy of calcined clay is primarily due to the calcination process, some studies indicate that it could be further reduced. The primary issue is a lack of sufficient or accurate data for calcined clay. However, all alternative mixtures demonstrated a reduction in environmental impact compared to mix BD435.

#### Material cost

The material cost for the unit-volume of concrete calculated and given in Table 3.

The contribution from the clinker phase to the overall cost was found to be significant and hence it is clear that blended mixtures were more economical than 100% Portland cement mixtures.

### Overall performance

Several parameters were discussed in the preceding sections, along with their individual contributions to each mixture. However, the overall performance of each mixture in terms of mechanical properties, environmental impact, and material cost must be discussed collectively. Therefore, each mixture should be ranked according to its sustainability. As mentioned in Sect. 2, two distinct approaches to analysing sustainable performance were considered.

**Fig. 6:**
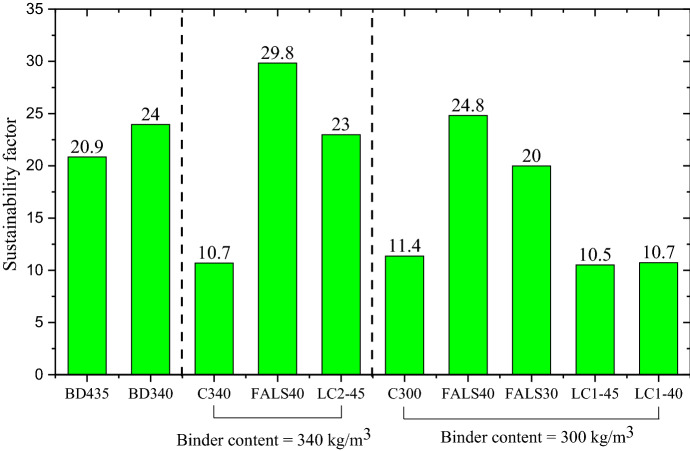
Sustainability factor of each mixture considering compressive strength, service life, and GWP

The sustainability factor for each mixture is depicted in Fig. 6 using Eq. 5. Increased values denote a more sustainable mixture. The highest value was obtained for FALS40 with a binder content of 340 kg/m^3^, while the lowest values were obtained for C300 and C340. Considering overall performance, the higher binder content with alternative binders were better than BD435.

**Fig. 7:**
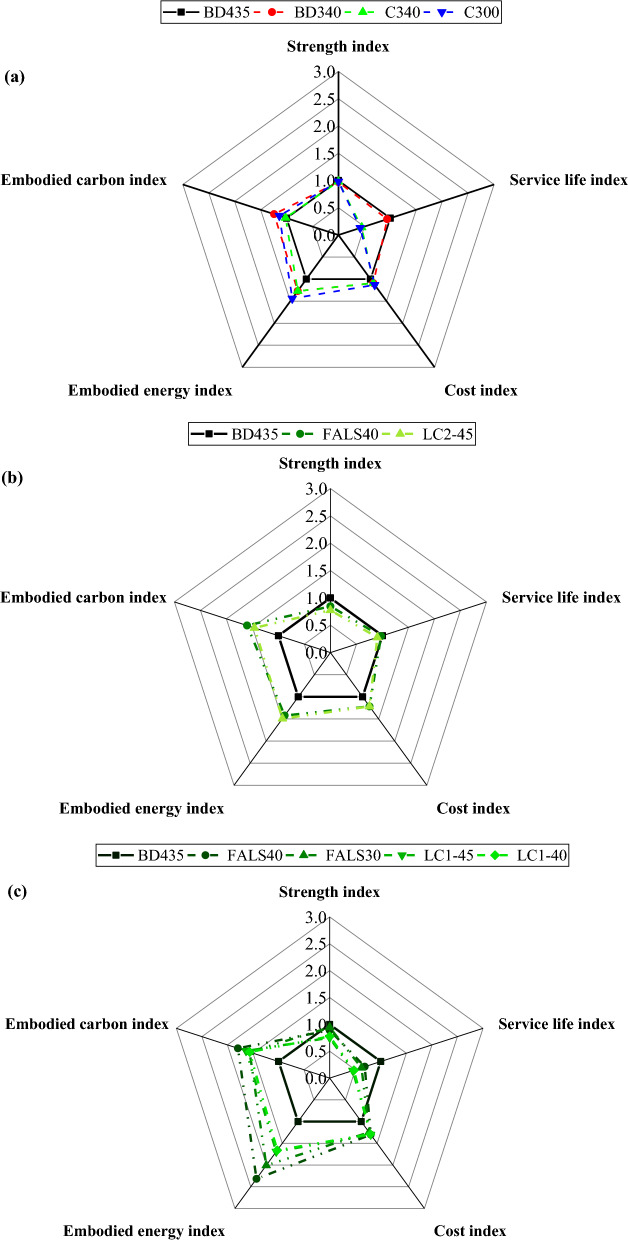
Comparison of individual indices of all mixes **a** BD435 versus control mixes with lowering cement content **b** BD435 versus alternative binder content = 340 kg/m^3^, and** c** BD435 versus alternative binder content = 300 kg/m^3^

**Fig. 8:**
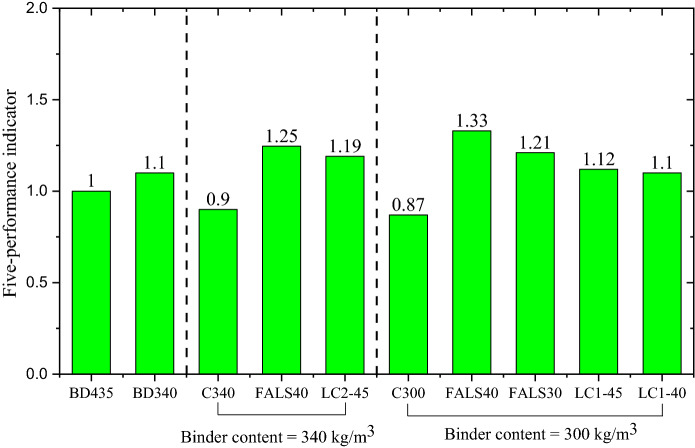
Five-performance indicator of all the mixture compared BD435 as reference mixture

In this technique, all sustainability parameters were weighted equally. In a concrete construction, for instance, service life is more significant than strength, particularly if the same desired compressive strength is achieved. On the other hand, a stronger concrete does not always imply more durable concrete across all environments. Simultaneously, not all concrete with superior durability must be high strength. Strength should be specified in terms of the structure’s ability to withstand applied loads, not as a performance metric for durability. As a result, the sustainability approach should be weighted differently and include additional parameters such as material cost. As a result, the second approach would provide a more precise picture of sustainability. Section 2 explained about the five-performance indicator and the distribution of each index illustrate each parameter’s performance. The *I*_5*P*_ values determined with BD435 as reference mixture are shown in the Fig. 7 and overall performance indicator illustrated in Fig. 8. Here also, a higher value of index is attributed to the better performing mix for the specific property of this index.

In the Fig. 7a, the individual indices of BD435 (the original bridge mix design), BD340, C340, and C300 are shown. From this first image it is visible that by just lowering cement content, higher indices connected to environmental impact can be achieved. However, there is a tipping point, at which the mix is increasing the indices connected to environmental impact, but at the same time with the sacrifice of service life, therefore having lower overall performance indicator (shown in Fig. 8). In the case of alternative binders with a total binder content of 340 kg/m^3^ (Fig. 7b), the significant reduction in the cement content and at the same time better durability (service life) was possible, leading to similar mechanical and service life indices and higher environmental indices. Overall performance indicator was therefore higher for mixes with alternative binder in total amount of 340 kg/m^3^, compared to that of original bridge mix design. Finally, when considering mixes with a total binder content of 300 kg/m^3^, compared to the original bridge mix design, it is evident that a limit in performance was reached. While alternative mixes do reach significantly higher environmental impact indices, the performance (mechanical and durability) are impaired. However, since all indices were considered with the same significance, all mixture performed significantly better than BD435, with the exception of the control mixtures in Fig. 8.

In comparison to the first method, the calcined clay mixture with both binder content levels showed good performance when compared to the bridge mixture. Even though the LC mixture demonstrated poor performance in terms of service life, they were still successful in achieving good sustainability in terms of environmental impact and materials cost. In the case of fly ash mixture, both mixes were significantly better than BD435.

Comparison of overall performance indicators shows how by decreasing the amount of cement used alone cannot result in an overall better performance, as was demonstrated by mixes C340 and C300. But reducing the amount of cement in the mixture and supplementing it with other cementitious materials that are locally available could produce mixtures with competitive sustainability performance indicators. Therefore, all alternative systems with reduced total binder have exhibited very good potential in the practical application. Additional to this, this study proved that good quality concrete can be produced with very low kaolin clay, producing concrete with comparable performance in terms of strength and durability compared to cement with slag or fly ash. Therefore, the performance-based design by optimisation of binder content and usage of alternative binder would be a good strategy for a more sustainable approach in concrete industry.

## Conclusion

In this experimental study, a real-structure concrete mix design was compared to different approaches of lowering carbon footprint of concrete. The concrete was prepared with reduced binder contents of 340 kg/m^3^ and 300 kg/m^3^. Next, the Portland cement was replaced by 30% and 45% with fly ash/calcined clay and limestone. The compressive strength and non-steady state chloride migration coefficients were evaluated for all the mixtures. From these results, the service life, environmental impact, and materials cost were evaluated. The overall sustainability of each mixture was evaluated and discussed using two different approaches.

Results presented in the study demonstrated on a real case scenario that the total binder content of concrete could be significantly reduced without compromising the mechanical and durability performance of concrete used in this specific structure exposed to harsh marine environment. Additionally, the usage of alternative binders along with reduction in the total binder content, provides an extra pull towards sustainability. The fly ash used, which was collected as waste stream, showed good results compared to the bridge mixture even with low binder content. The calcined clays used in this study was technically low-grade clay, and still it provided satisfying results in the overall sustainability performance. Unlike the fly ash and limestone mixture, in the case of calcined clay and limestone mixture, the kaolinite content and total binder significantly influenced the individual parameters. However, the five-performance sustainability approach demonstrated that low grade clay could also be a viable alternative to the original bridge design.

The study demonstrates unequivocally that optimizing total binder content and utilizing alternative binders (particularly those that are locally available) are critical factors in ensuring the overall sustainability of concrete. The study also experimentally proves that a significant savings in carbon footprint can be achieved without compromising mechanical stability or service life of concrete in structures even when exposed to aggressive marine environment.

## Supplementary Information

Below is the link to the electronic supplementary material.

Supplementary file1 (DOCX 30 kb)
