# Supplementary material for: Does carbon footprint reduction impair mechanical properties and service life of concrete?
Source: Mater Struct. 2022 Dec 29;56(1):6. doi: 10.1617/s11527-022-02090-9 (PMC9800350; doi:10.1617/s11527-022-02090-9)
Supplement: Supplementary file 1 — Supplementary file1 (DOCX 30 kb) [file 11527_2022_2090_MOESM1_ESM.docx]

**Additional information**

**Table 1 Chemical composition (oxides) of each binder material and particle mean size**

| Component | CEM I | CEM II | Fly ash (FA) | Calcined  clay 1 (CC1) | Calcined clay 2 (CC2) | Limestone (LS) |
| --- | --- | --- | --- | --- | --- | --- |
| CaO | 63.19 | 46.94 | 11.52 | 2.57 | 2.17 | 71.59 |
| SiO_2_ | 19.51 | 33.65 | 53.28 | 63.70 | 62.41 | 20.21 |
| Al_2_O_3_ | 4.21 | 7.55 | 19.11 | 19.53 | 21.35 | 4.32 |
| Fe_2_O_3_ | 2.85 | 2.38 | 9.05 | 6.80 | 7.26 | 1.43 |
| MgO | 0.85 | 3.39 | 2.78 | 2.34 | 1.78 | 1.69 |
| Na_2_O | 0.20 | 0.78 | 0.26 | 1.26 | 1.05 | 0 |
| K_2_O | 0.48 | 0.74 | 1.51 | 2.52 | 2.50 | 0.15 |
| TiO_2_ | 0.12 | 0.22 | 0.52 | 0.76 | 0.94 | 0.52 |
| P_2_O_5_ | 0.45 | 0.01 | 0.36 | 0.35 | 0.36 | 0.42 |
| SO_3_ | 2.3 | 4.02 | 1.48 | 0.12 | 0.07 | 1.48 |
| D_10_, μm | 0.32 | 0.45 | 1.55 | 3.13 | 4.03 | 3.43 |
| D_50_, μm | 9.95 | 12.71 | 15.24 | 12.16 | 10.72 | 18 |
| D_90_, μm | 50 | 58.85 | 72.68 | 42.43 | 24.79 | 63.82 |

**Figure 1 Particle size distribution of each binder materials**

**Table 2 Mix design**

| Mixture | Binder kg/m^3^ | w/b | Water, kg/m^3^ | Cement, kg/m^3^ | FA kg/m^3^ | CC kg/m^3^ | L kg/m^3^ | SP^*^ | Aggregate, kg/m^3^ | | |
| --- | --- | --- | --- | --- | --- | --- | --- | --- | --- | --- | --- |
|  |  |  |  |  |  |  |  | % | 16 – 8 | 8 – 4 | 4 – 0 |
| BD435 | 435 | 0.39 | 169 | 435 | - | - | - | 0.7 | 470 | 472 | 934 |
| BD340 | 340 |  | 132 | 340 | - | - | - | 1.2 | 518 | 520 | 1029 |
| C350 | 350 | 0.40 | 140 | 350 | - | - | - | 0.7 | 510 | 512 | 1014 |
| C340 | 340 |  | 136 | 340 | - | - | - | 0.8 | 513 | 514 | 1018 |
| C300 | 300 |  | 120 | 300 | - | - | - | 1.1 | 536 | 538 | 1064 |
| C250 | 250 |  | 100 | 250 | - | - | - | 2.1 | 561 | 563 | 1115 |
| C200 | 200 |  | 80 | 200 | - | - | - | 2.8 | 586 | 588 | 1165 |
| FA40 | 340 |  | 136 | 204 | 91.12 | - | 46.92 | 1.2 | 505 | 507 | 1003 |
| LC2-45 | 340 |  | 136 | 187 | - | 102 | 51 | 1.4 | 504 | 505 | 1001 |
| C300 | 300 | 0.45 | 135 | 300 | - | - | - | 1.0 | 525 | 527 | 1043 |
| C250 | 250 |  | 135 | 250 | - | - | - | 1.9 | 538 | 539 | 1066 |
| FA40 | 300 |  | 135 | 180 | 80.4 | - | 39.6 | 1.2 | 515 | 517 | 1023 |
| FA30 | 300 |  | 135 | 210 | 60.3 | - | 29.7 | 1.3 | 517 | 519 | 1028 |
| LC1-45 | 300 |  | 135 | 165 | - | 90 | 45 | 1.4 | 516 | 518 | 1025 |
| LC1-40 | 300 |  | 135 | 180 | - | 80.4 | 39.6 | 1.3 | 516 | 518 | 1025 |

FA – fly ash, CC – calcined clay, L – limestone, SP = Superplasticizer (amount expressed as mass percentage of total binder)

**Table 3 Embodied carbon and energy of ingredients in the mixture**

| **Material** | **Embodied carbon, (kg eq. CO_2_/ kg)** | **Embodied energy, MJ/kg** | **Reference** |
| --- | --- | --- | --- |
| CEM I 42.5R | 0.803 | 5.05 | [42] |
| CEM II B-S | 0.646 | 4.5 | [43] |
| LC2 blend | 0.210 | 3.04 | [12] |
| Fly ash | 0.008 | 0.1 | [44] |
| Limestone powder | 0.0172 | 0.35 | [45] |
| Crushed aggregate | 0.00702 | 0.113 | [45] |
| Superplasticizer | 1.88 | 4.8 | [46] |

**Table 4 Material cost of each ingredient**

| Material | Price in Euro/kg of material | Reference |
| --- | --- | --- |
| CEM I 42.5R | 0.099 | Local supplier |
| CEM II B-S | 0.093 |  |
| LC2 blend | 0.008 | [47] |
| Fly ash-limestone blend | 0.045 |  |
| Crushed aggregate | 0.00997 | Local supplier |
| Superplasticizer | 1.4 |  |
